# Estimation of parameters for a humidity-dependent compartmental model of the COVID-19 outbreak

**DOI:** 10.7717/peerj.10790

**Published:** 2021-02-18

**Authors:** Csaba Farkas, David Iclanzan, Boróka Olteán-Péter, Géza Vekov

**Affiliations:** 1 Mathematics and Computer Science, Sapientia Hungarian University of Transylvania, Targu Mures, Romania; 2 Mathematics and Computer Science, Babes-Bolyai University of Cluj-Napoca, Cluj-Napoca, Romania

**Keywords:** COVID-19, Mathematical model, Parameter estimation SARS-nCoV-2, Data fitting, PSO algorithm

## Abstract

Building an effective and highly usable epidemiology model presents two main challenges: finding the appropriate, realistic enough model that takes into account complex biological, social and environmental parameters and efficiently estimating the parameter values with which the model can accurately match the available outbreak data, provide useful projections. The reproduction number of the novel coronavirus (SARS-CoV-2) has been found to vary over time, potentially being influenced by a multitude of factors such as varying control strategies, changes in public awareness and reaction or, as a recent study suggests, sensitivity to temperature or humidity changes. To take into consideration these constantly evolving factors, the paper introduces a time dynamic, humidity-dependent SEIR-type extended epidemiological model with range-defined parameters. Using primarily the historical data of the outbreak from Northern and Southern Italy and with the help of stochastic global optimization algorithms, we are able to determine a model parameter estimation that provides a high-quality fit to the data. The time-dependent contact rate showed a quick drop to a value slightly below 2. Applying the model for the COVID-19 outbreak in the northern region of Italy, we obtained parameters that suggest a slower shrinkage of the contact rate to a value slightly above 4. These findings indicate that model fitting and validation, even on a limited amount of available data, can provide useful insights and projections, uncover aspects that upon improvement might help mitigate the disease spreading.

## Introduction

In November 2019 the virus named SARS-CoV-2 appeared in Wuhan, the capital city of Hubei province, a metropolis with 11 million inhabitants. On January 22 2020 an outbreak took place, with a massive infection count, that was later declared as a pandemic by the World Health Organization (WHO).

Physicists, epidemiologists, and mathematicians are trying to model the evolution of the currently raging outbreak, considering various models from basic propagation ones like SIR, IDEA to more sophisticated models like SEIR, SEIRS, statistical mechanics of open systems and the extended versions of the above.

In the early stages of an epidemiological outbreak, like the COVID-19 pandemic, when the available data is limited both in breadth and scope, many studies can only concentrate on model proposal (Tang et al., 2020a, 2020b; Zhou et al., 2020; Lin et al., 2020) and must omit the model fitting procedure and validation. However, as data becomes available, estimating the correct parameters is equally important in the case of epidemiological modeling, where the main criteria for explanatory power are the predictive power and falsifiability of the model. An inappropriate model will perform poorly even with the best possible parameter settings, same way as the the most appropriate model with badly chosen parameter values. Newer studies, such as Zhou et al. (2020), Liu et al. (2020a), Neto et al. (2020), and Kozio, Stanisawski & Bialic (2020), validated the proposed modells on data, in order to verify the parameter estimations and prediction of the trend of SARS-CoV-2 infections.

As it has been noticed before, some of the parameters of an epidemiological model are not well described by a single value, as these evolve and change over time. Thus, for the model to be able to capture these changes, one needs to consider the parameters as functions of time *t*. Recently, several articles (Tang et al., 2020b, 2020c; Zhou et al., 2020) proposed such models.

At the same time, the transmission rate in an outbreak can also change, it can be affected by climate conditions (such as temperature and humidity) (Lowen et al., 2007; Shaman et al., 2010; He et al., 2013). To the best of our knowledge, for the SARS-CoV-2 outbreak, there are only a few studies which take into account the weather conditions (Shi et al., 2020; Ma et al., 2020; Luo et al., 2020; Wang et al., 2020). Note that, the article Wang et al. (2020) uses non-epidemiological model to support its results,

Therefore, our first goal is to propose a general SEIR-based model (see “An Epidemiological Model for the SARS-CoV-2 Outbreak”) which incorporates biological, social and environmental processes that could account for temporal changes in transmission rate, such as governmental actions (school closing; Wu et al., 2010), weather changes (recent studies indicates temperature and humidity play a significant role in influenza transmission) and human behavioral responses (Caley, Philp & McCracken, 2008).

The second aim of the present article is to identify methods suitable for exact parameter estimation, even for complex models such as the one proposed in this study. Conceptually, the parameter estimation can be formulated as an optimization problem, where one wishes to find the parameter values that minimize the difference between the model output and the available empirical data. The parameters of the model are estimated in such way to minimize the difference between the model output and the available empirical data. However, this fitting procedure often proves difficult as traditional gradient based strategies such as Gauss–Newton methods (Nocedal & Wright, 2006), the Nelder–Mead (Nelder & Mead, 1965) or Hessian-free optimization algorithms like the Truncated Newton method (Nocedal & Wright, 2006; Nash, 2000; Martens, 2010) which quickly and easily get stuck in local optima, at sub-optimal parameter settings. Typically the number of local optima grows exponentially with system size, and becomes enormous for a model with 25 free parameters, as the one proposed in this paper (see “An Epidemiological Model for the SARS-CoV-2 Outbreak”). A random-restart gradient-based descent algorithm would need a proportional number of restarts, making the method unfeasible.

Therefore, we need a method that can escape local-optima and enables a better exploration and exploitation of the search space. In this article, we propose an iterated Particle Swarm Optimization (Kennedy & Eberhart, 1995) method (IPSO) where in each iteration the method explores only the vicinity of the best parameter estimation found previously. This approach significantly reduces the search space at each step and results in a gradual improvement process, where the method manages to advance to a significantly better local optima in almost every iteration. The gradual improvement seems to be enabled by the so called “big-valley” structure (Reeves, 2007) of the optimization problem, where local optima, good parameter estimations occur close to each other and a global optimum. IPSO can deliver a well-fitting parameter estimation for complex models, with more than 20 parameters, with a few hours of compute-time on a single processor.

Finally, in order to make this study available for everyone, we have developed a tool (https://seir-visualisation.vercel.app/) that visualizes the output of the proposed model. Therefore, visualization is also a significant part of our work. The visualization dashboard’s aim is to expand our limited study for uncountable cases with freely adjustable input. We recognize that existing studies become obsolete quite fast due to the fact that conditions and circumstances regarding the pandemic change very drastically (https://www.the-scientist.com/features/why-r0-is-problematic-for-predicting-covid-19-spread-67690). A case study of the evolution of the pandemic in Italian regions enables a better understanding of the virus’s factors and properties. However, if we cannot easily employ the model to new countries and regions, a significant part of our work’s benefits is lost. We reckon that this tool is the adequate way to adapt our study to new processes.

## Overview of infectious disease modeling and the SARS-CoV-2 outbreak

Since Kermack, McKendrick & Walker (1927) introduced their so called SIR mathematical model (Susceptible-Infected-Recovered compartmental model), which is composed of three differential equations, that is,

(1) $$\left\{ {\matrix{ & {\displaystyle{{dS} \over {dt}} = f(S(t),I(t)) = - \beta \cdot S(t) \cdot I(t)} \hfill \cr & {\displaystyle{{dI} \over {dt}} = \beta \cdot S(t) \cdot I(t) - k \cdot I(t)} \cr & {\displaystyle{{dR} \over {dt}} = k \cdot I(t)} \cr } } \right..$$

epidemiology started to make use of differential equation based models. These became one of the most powerful tools to determine infection counts and their evolution over time. Capasso & Serio (1978) generalized the original deterministic SIR model by replacing the linear interaction term with a nonlinear function which allows a more realistic and detailed control over the dependance on the number of infectious person. They point out that this way psychological effects can be taken into account, too.

A variant of the SIR model is the following so called SEIR model (for more information on the SEIR models, see Anderson & May (1992))

(2) $$\left\{ {\matrix{{\displaystyle{{dS} \over {dt}} = f(S(t),I(t))} \cr {\displaystyle{{dE} \over {dt}} = \beta \cdot S(t) \cdot I(t) - k \cdot E(t)} \cr {\displaystyle{{dI} \over {dt}} = k \cdot E(t) - \gamma E} \cr {\displaystyle{{dR} \over {dt}} = \gamma \cdot I(t)} \cr } } \right..$$

is to take into account the latent period ($\textstyle{1 \over k}$). For several infections there is a significant incubation period during which the individual has been infected but they are not yet infectious. Hence, the host cannot be categorized as susceptible, infectious, or recovered; we need to introduce a new category for these individuals who are infected but not yet infectious. These individuals are referred to as “exposed” (*E*). These type of models compute the theoretical number of people, sorting the cases into different groups, such as suspected cases, exposed cases, infectious cases, recovered cases. These epidemiological models are often used to model the spread of different diseases, such as the spread of smallpox on Easter Island in 1863 (Koss, 2019), of Severe Acute Respiratory Syndrome (SARS) (Naheed, Singh & Lucy, 2014) or Influenza A (H1N1) (Liu et al., 2015).

Further extensions occur when we assume that immunity lasts only for a limited period before the individual is once again susceptible (SIS, SEIRS model). For further reference, the book of Keeling & Rohani (2008) provides a more in-depth introduction to the modeling of infectious diseases. The authors start from the simplest of mathematical models and they show how the inclusion of appropriate elements of biological complexity leads to improved understanding of disease dynamics and control.

In Fisman et al. (2013), the authors proposes the *SIR* based *IDEA* (Incidence Decay and Exponential Adjustment) model, where the incidence count evolution prediction is based on the time-dependent decay of the reproduction number, adjusted by a discount factor, which is proposed based on the public health interventions and social concern. Despite its simplicity the model can be very efficient for small basic reproduction number, *R*_0_, but in case of constantly changing transmission and contact parameters, and complex public health control strategies it is less accurate.

Due to the urgency and the potentially devastating threat is poses, several studies already attempted to model and to predict the severity of COVID-19. In the following we summarize, without the sake of completeness, the growing literature about the SARS-CoV-2 outbreak. Studies use both basic and more advanced epidemiological models, that also incorporate a higher number of relevant parameters, such as medical case history, social and personal interactions, travel statistics, etc. While it is not possible to capture all aspects, the parameters that heavily define the spreading of a virus, if possible, should be accounted for. A detailed enumeration of such parameters can be found in Moore et al. (2016).

Commonly used epidemiological models for SARS-CoV-2 outbreak, are the above mentioned modified SIR, and SEIR-type models. Some studies, that simulate the geographical movement of people in the vicinity of the outbreak, sustain the parameter values of the basic epidemiological model. Usually, it is important to approximate the model’s parameters correctly, to fit to the measured data in order to increase the accuracy of the model as much as it is possible.

Tang et al. (2020a) presented one of the first mathematical models (SEIR-type model) for the novel coronavirus SARS-CoV-2 outbreak. In this article the authors use beside the usual categories (susceptible *S*, exposed *E*, infectious with symptoms *I*, recovered *R*) several other compartments, which are crucial from the modeling point of view, that is, they uses a separate compartment for the infectious but not yet symptomatic which is denoted by *A*, and also a different category is the number of hospitalized individuals, which is denoted by *H*. As a results of measures taken to reduce the spread of the disease the following compartments are also included in their model: quarantined susceptible, denoted by *S*_*q*_, isolated exposed *E*_*q*_ and finally isolated infected *I*_*q*_. The model’s advantage is that it captures the isolation and its effects, which is one of the most important tools to decrease the spreading of a disease. The methods used to approximate the model parameters were Markov Chain Monte Carlo (MCMC) and Metropolis–Hastings (M–H). The algorithms run more than 70,000 iterations, the prediction has 1,622−12,512 absolute error by the 7-th day depending on what type of contact rate it is used (no reduced contact, reduced contact by 50%, reduced contact by 90%). This compares with a daily average of 231.71–1,787.42, *which means that the infectious cases per day prediction’s errors can be significant*.

The above mentioned article was recently updated by Tang et al. (2020b). More precisely, the authors considered a time-dependent dynamic model which is built on the principles that different injunctions can significantly contribute to decreasing the contact rate *c* among persons, and the time period of diagnosis became shorter due to the increasing numbers of SARS-CoV-2 testing kits. This model describes more accurately the real-world situation, however, in the study only three parameters were estimated, the other ones were adopted from previous papers, which did not provide a good fit to data.

In Lin et al. (2020) a model is proposed, that extends the basic *SEIR* model by modeling the public perception of risk regarding the number of severe and critical cases and deaths $\left( D \right)$, cumulative cases (reported and not reported as well) $\left( C \right)$. The paper considered the transmission rate as a time-dependent function, which incorporates the impact of governmental action. This model also takes into account the number of individuals who leave Wuhan before the lock-down.

Other studies, such as Tang et al. (2020c), Zhou et al. (2020) also use similar epidemiological models. The Tang et al. (2020c) article uses time-dependent functions for contact rate, quarantined rate of exposed individuals, which incorporate prevention and control strategies, resulting in more punctual estimation. In Zhou et al. (2020), the authors investigate the effect of media, which brings a new equation into the system, named *M*, which represents the cumulative density of awareness programs driven by the media reports. This study used only a simple linear regression method.

In Wu, Leung & Leung (2020) the epidemics was simulated with help of SEIR-type epidemiological model. They studied the disease in Wuhan and the public health risk of pandemic based on the traveling in and out of the city. In Dolbeault & Turinici (2020) the authors studied a variant of the SEIR models to interpret some qualitative features of the statistics of the COVID-19 epidemic in France. The authors observed that after the lock-down, the social distancing is not enough to control the outbreak. A possible explanation for this issue, is that the lock-down is creating social heterogeneity. In He, Tang & Rong (2020) discrete-time stochastic epidemic model with binomial distributions was used to study the transmission of the disease. In order to calculate the total number of the infected population and the total number of fatality in Carcione et al. (2020) the authors implemented a new SEIR-type model. Taking into account the lack of suitable data and uncertainty of the different parameters they avoided the rigorous case study etc.

In Giordano et al. (2020) it is pointed out that to end the global SARS-CoV-2 pandemic multiple population-wide strategies have to be implemented, including social distancing, testing and contact tracing. They proposed a SEIR-type model as well which discriminates between infected individuals depending on whether they have been diagnosed and on the severity of their symptoms. This distinction is an important issue because the isolated individuals typically do not spread the virus.

In Liu et al. (2020b) authors use two epidemiological models to study the importance of the latency period. In order to validate their results they fit the models’ parameters to empirical data. For similar approach see also López & Rodo (2020). Later, in Liu et al. (2020c) the authors developed a *SEIR–type* model of COVID-19 epidemics to project the spread of the virus based on the early reported cases.

In Griette, Liu & Magal (2020) the authors developed a mathematical model to predict a bound for the ending date of the COVID-19 epidemics in mainland China with strong quarantine and testing measures for a sufficiently long time.

All these models are useful in highlighting critical factors, opening them up for discussions and analysis, and often narrowing down their distribution. However, without exact parameter estimations and at least a rough fit to available data, it is hard to objectively compare different models.

The study of Boldog et al. (2020) analyzes the situation from the other perspective using a complex system of three different models. The probabilistic system analyzes the effect of each individual imported case from importation until extinction. The first model in the system is a *SE*_2_*I*_3_*R* model which is providing *C* (cumulative number of cases). This result is used to find importation ratio in a country which is having θ connectivity and *C* imported cases from China and a baseline local reproductive number *R*_*loc*_. The effective infection scenario is modeled through a Galton–Watson branching process evaluating the branches, thus finding the value of the potential risk of an outbreak in the destination country. The findings show that, based on θ and *R*_0_ values, what is the optimal direction for the given country to minimize the risk of an outbreak. Countries with high θ and low *R*_0_ should lower their reproductive number, else if basic reproductive number is high and connectivity with China is low, then they should work on lowering even more the connection rate through screenings, even bans.

The effectiveness of traveling bans and it’s effect on possible outbreaks outside the base area is discussed in several articles. In a very recent article (Chinazzi et al., 2020), the authors analyze the travel restrictions implemented in Wuhan using the *GLEAM* (Global Epidemy and Mobility Model), optimizing it’s performance with Approximate Bayesian Computation. Their method approximates the posterior distribution of the basic reproductive number *R*_0_, analyzing the risk of importation of cases in other areas than Wuhan. The findings suggest that even when restricting the travel by 90%, as the case in Wuhan, with some latency, other outbreaks may possibly occur. The model also uses a transmission reduction factor *r*, which is representing the control actions for reducing the transmission rates, which are shown to be more effective than the travel restrictions.

**Figure 1:**
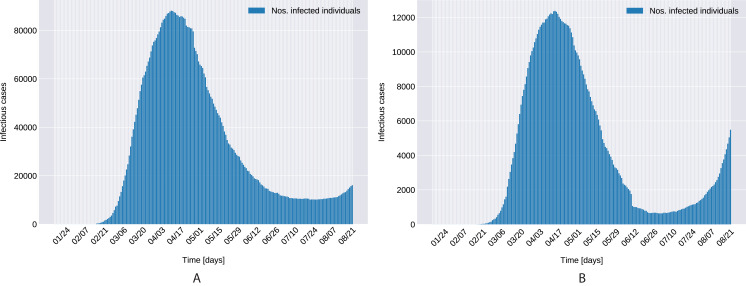
Daily number of reported infectious cases in Northern (A) and Southern (B) Italy.

Figure 1 presents the evolution of the daily number of reported infectious cases in North and South Italy, for the 24/01/2020–24/06/2020 period. The trend suggests that Italy managed to successfully curb the contact rate and effectively restrict the spreading of the infection. This statement can be reconciled with a recent study Li et al. (2020c), where the authors’ findings indicate that a radical increase in the identification and isolation of currently undocumented infections would be needed to fully control the novel coronavirus SARS-CoV-2.

## Materials and Methods

### An epidemiological model for the SARS-CoV-2 outbreak

Using epidemiological models, scientists have established a good understanding of the spread and control of infectious diseases from both mathematical and ecological points of view (Anderson & May, 1992; Kermack, McKendrick & Walker, 1927; Keeling & Rohani, 2008) (see also reference therein).

As seen in (1) and (2) the classical Susceptible-Infected-Recovered compartmental models assume that disease transmission is β·*SI* (where β, *S* and *I* denotes the transmission rate, the number of susceptible and infectious individuals, respectively). During the study of the cholera epidemic spread in Bari, Capasso & Serio (1978) observed that the incidence rate may increase more slowly as *I* increases rather than linearly, if we use the following non-linear function $f(S,I) = \textstyle{{\beta SI} \over {1 + {k_1}I}},\;{k_1} > 0$. After their study, several nonlinear functions were used in epidemic models, for more details see Adnani, Hattaf & Yousfi (2013), Kaddar (2009), Zhou, Xiao & Li (2007) (see also reference therein).

This observations opened new directions in the epidemiological modeling, that is, several authors used some new sophisticated non-linear functions to describe the modeled phenomena more accurately, see for instance Lahrouz et al. (2012), Xiao & Ruan (2007), Cui, Sun & Zhu (2008). For example, in Cui, Sun & Zhu (2008), the authors used the non-linear function *βe*^−*mI*(*t*)^*S*(*t*)*I*(*t*) (which is a non-monotonic and non-concave function) to incorporate the media impact in their model.

In addition to the use of new non-linear functions, the following question can be formulated: in a very general case what assumptions on the function *f* should guarantee a global stability result for the proposed model?

A possible answer to this question can be found in Wang, Zhang & Liu (2018), (see also Xiao, Tang & Wu (2015)) where the authors considered a very general non-linear *f*(*S*, *I*) with the following assumptions:

(*f*_1_) *f*, *f*_1_:[0, + *∞*)× [0, + *∞*)→[0, + *∞*), with *f*(0, *I*) = *f*(*S*, 0) = 0 and *f*(*S*, *I*) = *If*_1_(*S*, *I*);
(*f_2_)* $\displaystyle{{\partial {f_1}(S,I)} \over {\partial S}} \gt 0$, and $\displaystyle{{\partial {f_1}(S,I)} \over {\partial I}} \le 0$ for all *S*, *I* ≥ 0.

With the help of the above assumptions, in Wang, Zhang & Liu (2018) a global stability can be found.

Taking into account the above studies, we propose the following SEIRS-type model, which builds upon the models from Tang et al. (2020a) and Xiao, Tang & Wu (2015):

(3) $$\left\{ {\matrix{ {{S}^{\prime} = - w(S,I,A) + \lambda {S_q}} \cr {{E}^{\prime} = f(S,I,A) - \sigma E} \cr {{I}^{\prime} = \sigma \rho E - \left( {{\delta _I} + \alpha + {\gamma _I}} \right)I} \cr {{A}^{\prime} = \sigma \left( {1 - \rho } \right)E - {\gamma _A}A + {\gamma _R}R} \cr {{S^{\prime}_q} = g(S,I,A) - \lambda {S_q}} \cr {{E^{\prime}_q} = h(S,I,A) - {S_q}{E_q}} \cr {{H}^{\prime} = {\delta _I}I + {\delta _q}{E_q} - \left( {\alpha + {\gamma _H}} \right)H} \cr {{R}^{\prime} = {\gamma _I}I + {\gamma _A}A + {\gamma _H}H - {\gamma _R}R} \cr } } \right.$$

where, as in the usual case, denote by *S*, *E*, *I* and *R* respectively be the proportion of the population susceptible, exposed, infectious, and recovered at time *t*. Similarly to Tang et al. (2020a), we also consider the following functions: denote by *A*(*t*) the number of pre-symptomatic cases, by *H*(*t*) the number of hospitalized cases in time *t*. Also denote by *S*_*q*_(*t*) the number of quarantined susceptible, the *E*_*q*_(*t*) isolated exposed, and *I*_*q*_(*t*) the number isolated infected compartments in time *t*. In such a general framework, we have that

$$w(S,I,A)=f(S,I,A)+g(S,I,A)+h(S,I,A)$$

The parameter σ and λ describes the transition rate of exposed individuals to the infected class and the rate at which the quarantined uninfected were released into the wider community, while the parameter ϱ represents the probability of having symptoms among infected individuals. The parameters δ_*I*_ and δ_*q*_ denote the transition rate of symptomatic infected and quarantined exposed to the quarantined infected class. The γ*_I_*, γ*_A_* and γ_*H*_ represent the recovery rate of symptomatic, asymptomatic and quarantined infected individuals, and finally γ_*R*_ is the rate at which immunity is lost and recovered individuals move into the pre-symptomatic class (according to a recent NHK-World Japan report and Li et al. (2020a)). We assume that natural birth and natural death rates are equal.

From their definitions, it follows that the models presented in Tang et al. (2020a) use the following functions:

(4) $$\left\{ {\matrix{ {w(S,I,A) = \left[ {\beta c + cq\left( {1 - \beta } \right)} \right]S\left( {I + \theta A} \right)} \cr {f(S,I,A) = \beta c\left( {1 - q} \right)S\left( {I + \theta A} \right)} \cr {g(S,I,A) = \left( {1 - \beta } \right)cqS\left( {I + \theta A} \right)} \cr {h(S,I,A) = \beta cqS\left( {I + \theta A} \right)} \cr } } \right.$$

The motivation of such a choice is the following (Castillo-Chavez, Castillo-Garsow & Yakubu (2003); Xiao, Tang & Wu (2015)): individuals move from quarantined cases with 1 − *q* proportion to *S*_*q*_ and with *q* proportion to *E*_*q*_. If the transmission probability is β and the contact rate is *c*, then, the infected quarantined individuals move to *E*_*q*_ at rate of β*cq* and uninfected quarantined individuals move to *S*_*q*_ at $\left( {1 - \beta } \right)cq$ rate. In case of not quarantined infected individuals, they are going to move to *E* at a rate of $\beta c\left( {1 - q} \right)$. The parameter θ represents the relative transmission probability of pre-symptomatic individuals to infected individuals.

In the hope of capturing more accurately the effect of factors that influences the outbreak progression, we choose the following functions in (3):

(5) $$\left\{ {\matrix{ {w(S,I,A) = \left[ {\beta (t)c(t) + c(t)q(t)\left( {1 - \beta (t)} \right)} \right]S\left( {I + \theta A} \right),} \cr {f(S,I,A) = \beta (t)c(t)\left( {1 - q(t)} \right)S\left( {I + \theta A} \right),} \cr {g(S,I,A) = \left( {1 - \beta (t)} \right)c(t)q(t)S\left( {I + \theta A} \right),} \cr {h(S,I,A) = \beta (t)c(t)q(t)S\left( {I + \theta A} \right).} \cr } } \right.$$

Such choice of functions *w*, *f*, *g*, *h*, incorporates biological, social or environmental processes that could account for temporal changes in transmission rate, in contact rate and in quarantined rate of exposed individuals.

When an epidemiological outbreak occurs, many preemptive actions can be taken to mitigate the spreading. Once people become informed, they can change their behavior, for instance, working from home, practicing social distancing and take actions such as often hand washing, wearing protective apparel, disinfecting etc., all of them contributing to the prevention of the spread. When the media interacts with the susceptible population, it starts influencing them to take appropriate measures to minimize the chances of getting infected. This media influence is initially low and increases as the infection increases. This observation suggests the following contact rate function:

(6) $$c(t) = {c_a} + \displaystyle{{3({c_0} - {c_a})} \over {1 + 2{b^{ - t}}}},\;t \ge 0$$

where *b* < 1 and *c*_0_ denotes the initial contact rate, while *c*_*a*_ denotes the minimum contact rate under the current control strategies. Thus, *c*(0) = *c*_0_, and 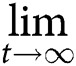
*c*(*t*) = *c*_*a*_

Control measures taken in case of possible outbreak include local and international traveling bans, isolation at residence, quarantine. To account for such countermeasures, we propose an increasing function *q*(*t*) for the quarantined rate of exposed individuals, defined by

$$q(t)=\frac{q_1t+q_0}{t+1},\ t\geq 0,$$

where at the initial quarantined rate is *q*(0) = *q*_0_, and the maximum quarantined rate under the current control strategies is 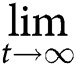
*q*(*t*) = *q*_1_.

Finally, for the transmission rate we propose the following function, which is defined by

$$\beta(t)=(1-\alpha_\beta)\beta_0\cdot(1+\xi AH(t))\cdot \left(1-\frac{I(t)+A(t)}{S(t)+R(t)}\right)^2, \ t\geq 0,$$

where *β*_0_ denote the baseline transmission rate, *AH*(*t*) denotes absolute humidity, ξ is the amplitude of the response of transmission rate to absolute humidity changes (recent studies indicates temperature and humidity play a significant role in influenza transmission (Lowen et al., 2007; Shaman et al., 2010; He et al., 2013; Wang et al., 2020); for COVID-19 see Ma et al. (2020), Shi et al. (2020), Luo et al. (2020)), α_β_ incorporates the impact of governmental action, while the last final factor represents human behavioral responses (for previous study see Caley, Philp & McCracken (2008)). Note that, for the absolute humidity (*AH*) we use the so called Clausius–Clapeyron equation (for more information see Iribarne & Godson (1973)), that is,

$$AH(t)={\frac{\displaystyle 6.112\cdot e^{\textstyle\frac{17.67\cdot T(t)}{\textstyle{T(t)+243.5}}}\cdot RH(t)\cdot2.1674} {273.15 +T(t)}},$$

where *T*(*t*) denotes the temperature in time *t*, while *RH*(*t*) denotes the relative humidity in percent (0 − 100)

Using the method of next generation matrix (Diekmann, Heesterbeek & Metz, 1990; Van den Driessche & Watmough, 2002; Tang et al., 2020a) one can define the effective daily reproduction number as

(7) $$R(t) = \left[ {\displaystyle{{\rho \beta (t)c(t)(1 - q(t))} \over {{\delta _I} + \alpha + {\gamma _I}}} + \displaystyle{{\theta (1 - \rho )\beta (t)c(t)(2 - q(t))} \over {{\gamma _A}}}} \right]{S_0}.$$

Note that, *R*(*t*) is the number of new infections by a single infected individual during his infectious period per day. Thus, form (7) one has that

$$R(t)=\beta(t)c(t)(1-q(t))\left[\frac{\rho}{\gamma_{I}+\delta_I}+\frac{\theta(1-\rho)}{\gamma_A}\right]S_0.$$

Now, denoting by, $M = \left( {\displaystyle{\rho \over {{\gamma _I} + {\delta _I}}} + \displaystyle{{\theta (1 - \rho )} \over {{\gamma _A}}}} \right){S_0} > 0$, we get that *R*(*t*) = *Mβ*(*t*)*c*(*t*)(1 − *q*(*t*)), or equivalently

$$\frac{R(t)}{\beta(t)}=M\cdot c(t)(1-q(t)).$$

From the definition of the function *c*(*t*), it follows that

$$c_0\geq c(t)\geq \lim_{t\to \infty}c(t)=c_a.$$

In a similar way, one has that

$$q_0\leq q(t)\leq \lim_{t\to \infty} q(t)=q_1.$$

Combining the above outcomes, it follows that ${c_a}(1 - {q_1})M \le \displaystyle{{R(t)} \over {\beta (t)}} \le {c_0}(1 - {q_0})M,$ or

$$c_a(1-q_1)M\beta(t)\leq R(t)\leq c_0 (1-q_0)M \beta(t).$$

The above inequality together with the definition of the function β shows, that

$$R(t) \le {c_0}(1 - {q_0})M \cdot (1 - {\alpha _b}){\beta _0} \cdot \left( {1 + \xi \mathop {\max }\limits_t AH(t)} \right) \cdot \mathop {\max }\limits_t {\left( {1 - \displaystyle{{I(t) + A(t)} \over {S(t) + R(t)}}} \right)^2}$$

$${\hskip17pt}\le {c_0}(1 - {q_0})M \cdot (1 - {\alpha _b}){\beta _0} \cdot \left( {1 + \xi \mathop {\max }\limits_t AH(t)} \right).$$

In order to get an upper bound for *R*(*t*), we consider the function $f:[ - 20,50] \to {\mathbb{R}}$ defined by

$$f(x)=\frac{1}{x+273.15}\cdot e^{\frac{17.67\cdot x}{x+243.5}}.$$

Since *x* ≤ 50, it is easy to see that

$$f'(x) =  - {{{e^{{{17.67 \cdot x} \over {x + 243.5}}}}} \over {{{(x + 273.15)}^2}}} + {{\left( {{{17.67} \over {x + 243.5}} - {{17.67x} \over {{{(x + 243.5)}^2}}}} \right){e^{{{17.67 \cdot x} \over {x + 243.5}}}}} \over {x + 273.15}},$$

which means that the function *f* is an increasing function. Therefore

$$f(T_{\rm min})\leq f(T(t))\leq f(T_{\rm max}),$$

where *T*_max_ = max_*t*_*T*(*t*) and *T*_min_ = min_*t*_ *T*(*t*). Thus, combining the above outcomes, one can see that

(8) $${K_{{\rm m}in}} \le R(t) \le {K_{{\rm m}ax}},$$

where *RH*_max_ = max_*t*_*RH*(*t*), *RH*_min_ = min_*t*_ *RH*(*t*) and

$$\eqalign{K_{\rm max}:=&c_0 (1-q_0)M\cdot (1-\alpha_{b}) \cr& \beta_0 \cdot \left[1+\xi \left(6.112\cdot 2.1674 \cdot RH_{\mbox{max}}\cdot f(T_{\mbox{max}})\right)\right]\cdot \max_{t}\left(1-\frac{I(t)+A(t)}{S(t)+R(t)}\right)^2,}$$

and

$$\matrix{{{K_{{\rm{min}}}}: = } & {{c_a}(1 - {q_1})M(1 - {\alpha _b})} \cr {} & {{\beta _0}\left[ {1 + \xi \left( {6.112 \cdot 2.1674 \cdot R{H_{{\rm{min}}}} \cdot f({T_{{\rm{min}}}})} \right)} \right] \cdot \mathop {\min }\limits_t {{\left( {1 - {{I(t) + A(t)} \over {S(t) + R(t)}}} \right)}^2}.} \cr } $$

To understand how to control and liquidate infectious diseases is one of the main goals of mathematical epidemiology. We know that a disease can cause an epidemic if and only if the basic reproduction number (the expected number of secondary cases caused by a primary case in a fully susceptible population, denote by ${\bar {\cal R}}$) is greater than 1, Van den Driessche & Watmough (2002). In order to fight the currently ragging outbreak, we need to reduce ${\bar {\cal R}}$ to less than 1. According to He et al. (2013) and Ma & Ma (2006), stability of the system can be connected with the basic reproduction number ${\bar {\cal R}}$ of the time-average systems (replacing the time-varying parameters with their long-term time averages), that is,

$${\bar {\cal R}}=\hat{\beta}\cdot \hat{c}\cdot (1-\hat{q})\left(\frac{\rho}{\gamma_{I}+\delta_I}+\frac{\theta(1-\rho)}{\gamma_A}\right)S_0,$$

where $\hat g$ represents the time average of the function *g*, that is,

$$\hat{g}=\lim_{t\to \infty}\frac{1}{t}\int_0^t g(s)\mathrm{d}s.$$

By a simple calculation it yields that $\hat c = {c_a},$ and $\hat q = {q_1}$ (Fig. 11).

Thus,

$${\bar {\cal R}}=\hat{\beta}\cdot c_a\cdot (1-q_1)M.$$

The correlation provided by Wang et al. (2020) refers to this averaged reproduction number, and, if ${\bar {\cal R}}$ is numerically solvable, we are confident, that our model will show the same sensitivity to temperature changes as the measurements in the before-mentioned study. However, we can make the following estimate for ${\bar {\cal R}}$:

$$\hat \beta = \mathop {\lim }\limits_{t \to \infty } \displaystyle{1 \over t}\int_0^t \beta (s){\rm d}s \le (1 - {\alpha _b}){\beta _0} \cdot \mathop {\lim }\limits_{t \to \infty } \displaystyle{1 \over t}\int_0^t \left( {1 + \xi AH(s)} \right){\left( {1 - \displaystyle{{I(s) + A(s)} \over {S(s) + R(s)}}} \right)^2}{\rm d}s$$

$${\hskip83pt}\le (1 - {\alpha _b}){\beta _0}\left[ {1 + \xi \left( {6.112 \cdot 2.1674 \cdot R{H_{{\rm max}}} \cdot f({T_{{\rm max}}})} \right)} \right].$$

Thus,

$$\displaystyle {\bar {\cal R}}\leq \beta_0 M (1-\alpha_{b})\cdot c_a\cdot (1-q_1) \left[1+\xi \left(6.112\cdot 2.1674 \cdot RH_{\mbox{max}}\cdot f(T_{\mbox{max}})\right)\right].$$

Similarly as above,

$$\matrix{ {\bar{R} \ge \,{c_a}{\beta _0} \cdot (1 - {\alpha _b}) \cdot (1 - {q_1})M} \hfill \cr {\left[ {1 + \xi \left( {6.112 \cdot 2.1674 \cdot R{H_{{\rm{min}}}} \cdot f({T_{{\rm{min}}}})} \right)} \right] \cdot {{\min }_t}{{\left( {1 - {{I(t) + A(t)} \over {S(t) + R(t)}}} \right)}^2}.} \hfill  \cr } $$

In the rest of this section we deal with the initial values of the proposed model. Accordingly, all the initial values of the functions and parameters of the models are detailed in Tables 1 and 2. Since, the first case in Europe was confirmed in Bordeaux on 24 January, we use the following initial conditions *E* = *I* = *A* = *S*_*q*_ = *E*_*q*_ = *H* = *R* = 0, and *S* = 28,328,178 for Northern Italy and *S* = 17,757,000 for Southern Italy. For each parameter we provide the acceptable value ranges, and where available, we indicate the source of these.

**Table 1:** Initial conditions for the functions.

Functions	Definitions	Initial values (Northern Italy (N.I.) and Southern Italy (S.I.))
*S*	Susceptible population	N.I.:28328178, S.I.:17757000
*E*	Exposed population	0
*I*	Symptomatic infected population	0
*A*	Asymptomatic infected population	0
*S*_*q*_	Quarantined susceptible population	0
*E*_*q*_	Quarantined exposed population	0
*R*	Recovered population	0

**Table 2:** Initial values for the parameters.

Parameters	Definitions	Range	Source
*c*_0_	Initial contact rate	(12.48–55)	Tang et al. (2020c)
*c*_*a*_	Minimum contact rate under control strategies	(1.25–10)	
*q*_1_	Maximum quarantined rate under control strategies	(1.57*e*−12–1.57*e*−4)	Tang et al. (2020b)
β	Probability of transmission per contact	(1.57*e*−12–1.57*e*−4)	Tang et al. (2020b)
*q*_0_	Initial quarantined rate of exposed individuals	(5.631*e*−10 to 5.631*e*−2)	
σ	Transition rate of exposed individuals to the infected class	(1/15–1/3)	WHO
λ	Rate at which the quarantined uninfects were released into the wider community	−	WHO
ϱ	Probability of having symptoms among infected individuals	(0.6–0.98)	Tang et al. (2020b)
δ*_I_*	Transition rate of symptomatic infected individuals to the quarantined infected class	(0.01–0.95)	Tang et al. (2020a)
δ*_q_*	Transition rate of quarantined exposed individuals to the quarantined infected class	(0.01–0.4)	Tang et al. (2020b)
γ*_I_*	Recovery rate of symptomatic infected individuals	(0.01–0.75)	Tang et al. (2020b)
γ*_A_*	Recovery rate of asymptomatic infected individuals	(0.01–0.36)	Tang et al. (2020b)
γ*_H_*	Recovery rate of quarantined infected individuals	(0.01–0.2)	Tang et al. (2020b)
γ*_R_*	Rate at which recovered individuals move into the pre-symptomatic class	(3*e*−7 to *e*−4)	–
θ	Relative transmission probability of *A* compared with *I*	(*e*−7 to 0.1)	Zhou et al. (2020)
α*_b_*	Governmental action strength	(0–1)	Lin et al. (2020)
ξ	The intensity of the effect of temperature variation	(*e*−18 to 0.999)	He et al. (2013)
*b*	Coefficient for contact rate function	(65–1)	–

From all these parameters, only *S*_0_ and λ are kept fixed, all the others are approximated, starting from their initial values.

### Data

We obtained the data regarding the cumulative number of laboratory-confirmed COVID-19 cases, cumulative number of recoveries, cumulative number of deaths and cumulative number of hospitalized in Northern and Southern Italy from the dataset provided by Italian Department of Civil Protection (http://www.protezionecivile.it/web/guest/department). The database is continuously updated and made freely available trough a GitHub repository (https://github.com/pcm-dpc/COVID-19/blob/master/dati-regioni/dpc-covid19-ita-regioni.csv).

We accounted for Northern Italy the following provinces: Aosta Valley, Emilia-Romagna, Friuli-Venezia Giulia, Liguria, Lombardy, Piedmont Veneto, Trentino-Alto Adige and Tuscany. Southern Italy’s provinces are Abruzzo, Apulia, Basilicata, Calabria, Campania, Molise, Sicily, Sardinia.

Temperature and humidity data were retrieved from AccuWeather Inc. (https://www.accuweather.com/) monthly view/daily data and www.worldweatheronline.com’s API, taking each region center’s or city’s airport data, when available. In case of Northern Italy and Southern Italy the above listed regions’ values were averaged, this being used as daily temperature and humidity.

### Parameter estimation of the epidemiological model

In mathematical biology, modeling complex phenomena by ordinary differential equations pose among others, two main challenges: (i) finding the appropriate model with the right balance of complexity and explanatory power and (ii) estimating correctly the parameters of the selected model.

Model parameter estimation often proves difficult for large models, due the multiomdality and combinatorial explosion, induced by the many and non-linearly linked parameters.

More versatile optimization methods, like evolutionary algorithms and other meta-heuristics, are usually more successful but also computationally much more expensive as they search through the entire combinatorial parameter space.

Genetic Algorithms (GA) have been successfully utilized to optimize the parameters of epidemiological models for the Severe Acute Respiratory Syndrome (SARS) (Yan & Zou, 2008), cholera (Akman & Schaefer, 2015; Akman, Corby & Schaefer, 2016) and recently for the SARS-CoV-2 outbreak (Neto et al., 2020; Yousefpour, Jahanshahi & Bekiros, 2020; Kozio, Stanisawski & Bialic, 2020).

Recent studies (Akman, Akman & Schaefer, 2018; Putra & Khozin Mu’tamar, 2019) highlighted that Particle Swarm Optimization (PSO) (Kennedy & Eberhart, 1995) methods might be better suited to fit epidemiological models to data, outperforming evolutionary algorithms.

The PSO is a population based meta-heuristic where the potential solutions are called “particles” and the population of solutions forms a “swarm”. PSO roughly mimics the social behavior of birds flocking or fish schooling for food, hoping to capture the swarm intelligence emerging from the cooperation between individuals (Kennedy & Eberhart, 1995; Clerc, 2010). The method has a more stable convergence characteristics than other stochastic methods and it is easily parallelizable. Because the method does not rely on gradients, it is well-suited to optimizing non-linear or discontinuous systems (Jalilzadeh et al., 2009). This property makes it amenable for ODE model parameter estimation, where small parameter variations can result in significantly different prediction errors.

Solutions in PSO are encoded as *n*-tuples of real numbers. At the start of the method each particle is randomly placed in the *n* dimensional space. The methods’ core concept is that the particles are flown through ${\mathbb{R}}$ in discrete time steps, and at each iteration the particles are responding to the quality factors of the best solution, being accelerated towards them. PSO keeps track of the overall best value, solution seen so far and the actual best value, solution present in the swarm. At each iteration, acceleration toward these solutions is weighted by random terms, in order to enable a better exploration of the solution space.

#### Error function

As PSO is a Hessian-free optimization method, that does not require the derivative of the error function, we choose to satisfy the *L*_1_ norm optimality criterion, minimizing the sum of absolute errors.

Formally, we wish to:

(9) $$argmi{n_P}\sum\limits_{t = 0}^{D - 1} ||I(t) - (\bar C(t) - \bar R(t) - \bar D(t))||$$

where *P* is the parameter set of the model we want to estimate (some parameter values might be already known, have fixed values), *D* is the number of days for which we have data available, *I*(*t*) is the predicted number of infected people by our model on day *t*, $\bar C$, $\bar R$; and $\bar D$; are the reported numbers (data) for the cumulative confirmed cases, the number of cumulative recoveries, respectively the number of cumulative deaths by COVID-19.

**Figure 2:**
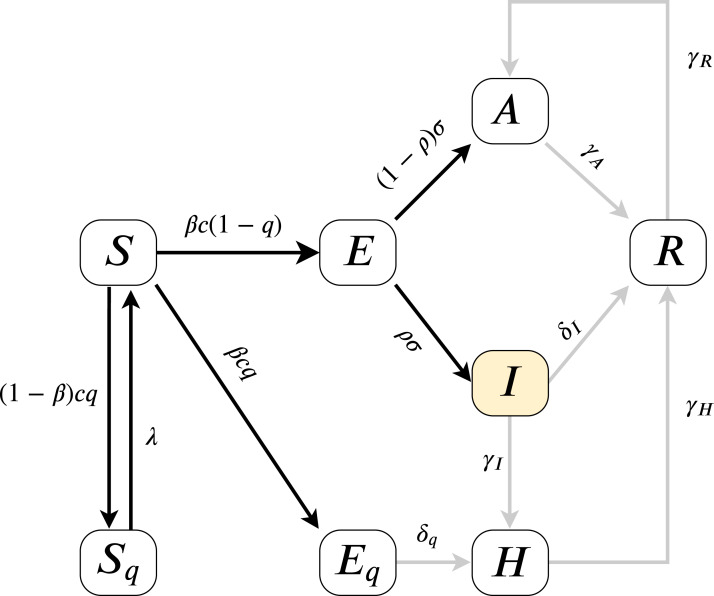
Model diagram for infection dynamics.

As the model fitting is based on measuring the error on *I*(*t*), we can estimate the parameters present on the highlighted, black paths from Fig. 2, the directed edges before *I*. All outgoing parameters from*I*can only be aggregately approximated (sum of δ_*I*_, γ_*I*_) and γ_*R*_, γ_*H*_ cannot be assessed. Therefore our analysis models $\beta (t),{\kern 1pt} c(t),{\kern 1pt} q(t),\sigma ,\rho$ (for more details of the parameters see “An Epidemiological Model for the SARS-CoV-2 Outbreak” and Table 2) and their effects on *I*(*t*). Since according to official numbers death cases are not divided into compartments from where the cases come (*H* or *I*), in this work we neglect death rate, we assume α = 0.

#### Iterative particle swarm optimization

The placement of local-optima in real-world optimization problems is usually not random, high quality solutions tend to be clustered close together (Reeves, 2007; Ochoa et al., 2011). Iterated local search Lourenço, Martin & Stützle (2019) methods exploit this structured distribution of local optima, advancing from one local optima to a better one with the help of random perturbations.

Therefore, we propose an iterated PSO method (IPSO) where the particles only explore the vicinity of an established good solution, greatly reducing the search space. Once the exploration around one solution has finished and a better local optima was identified, the method moves to explore the proximity of that solution.

**Algorithm 1:** Remapping of the particles to parameter estimations.

**Function** *remap(particle)* **is**
**Data:** *n* - number of parameters
**Data:** *s f* - scale factor used in remapping; default value is 2
**Data:** *params* - solution around which to perform the remap
**Data:** *L*, *U* - functions that return the lower, respectively the upper bound for a
parameter index from the model
**Data:** *clip*(*value*,*min*,*max*) - function that clips the input value between a
minimum and maximum value
**Result:** the remapped solution around *params* by using *particle*
*solution*←*empty*() ;
**for** *i*←1 **to** *n* **do**
*solution*[*i*]←*clip*(*params*[*i*] * *particle*[*i*] * *s f*,*L*(*i*),*U*(*i*)) ;
**end**
**return** *solution*;
**end**

Reduction of the solution space around one solution is achieved by

1. Restricting the PSO to a search on ${[0 \ldots 1]^n}$.
2. Using the particles, not as direct solution, but as scaling factors used to remap the solution whom neighborhood we are currently exploring, as presented in listing 1.

To minimize the compute time for the parameter estimation, the number of particles and number of generations per one PSO run is gradually increased each iteration. In this way, more compute resources are allocated in later phases, when the method has to improve on already good parameter estimations.

### Experiments

We estimated all relevant parameters of our model (see Fig. 2) based on the epidemiological, weather and humidity data for the Northern and Southern Italy for the interval 24/01/2020–23/05/2020 inclusive, totaling 120 days. The 99 day period of 24/05/2020–31/08/2020 inclusive was reserved for validation.

The initial conditions for the functions from Table 1 were chosen due to the fact that on 24 January, the first case in Europe was confirmed in Bordeaux (https://www.bbc.com/news/world-europe-52526554). Two more cases were confirmed in Paris by the end of the day, all of them originated from China. Until the first case in Northern Italy which was in 21/02/2020 (https://en.wikipedia.org/wiki/COVID-19_pandemic_in_Italy), we chose every initial value as 0.

For both parameter set estimations, the IPSO was run for 20 iterations. For each iteration *i*, the particle swarm size was set to 100 * *i* and the generation number to 5 * *i*.

## Online visualization dashboard

The dashboard is a serverless webpage, developed in Svelte (https://svelte.dev), that includes three main components. The first component is the data entry part, the second part concerns the data visualization, and lastly, the third one is a differential equation solver.

**Figure 3:**
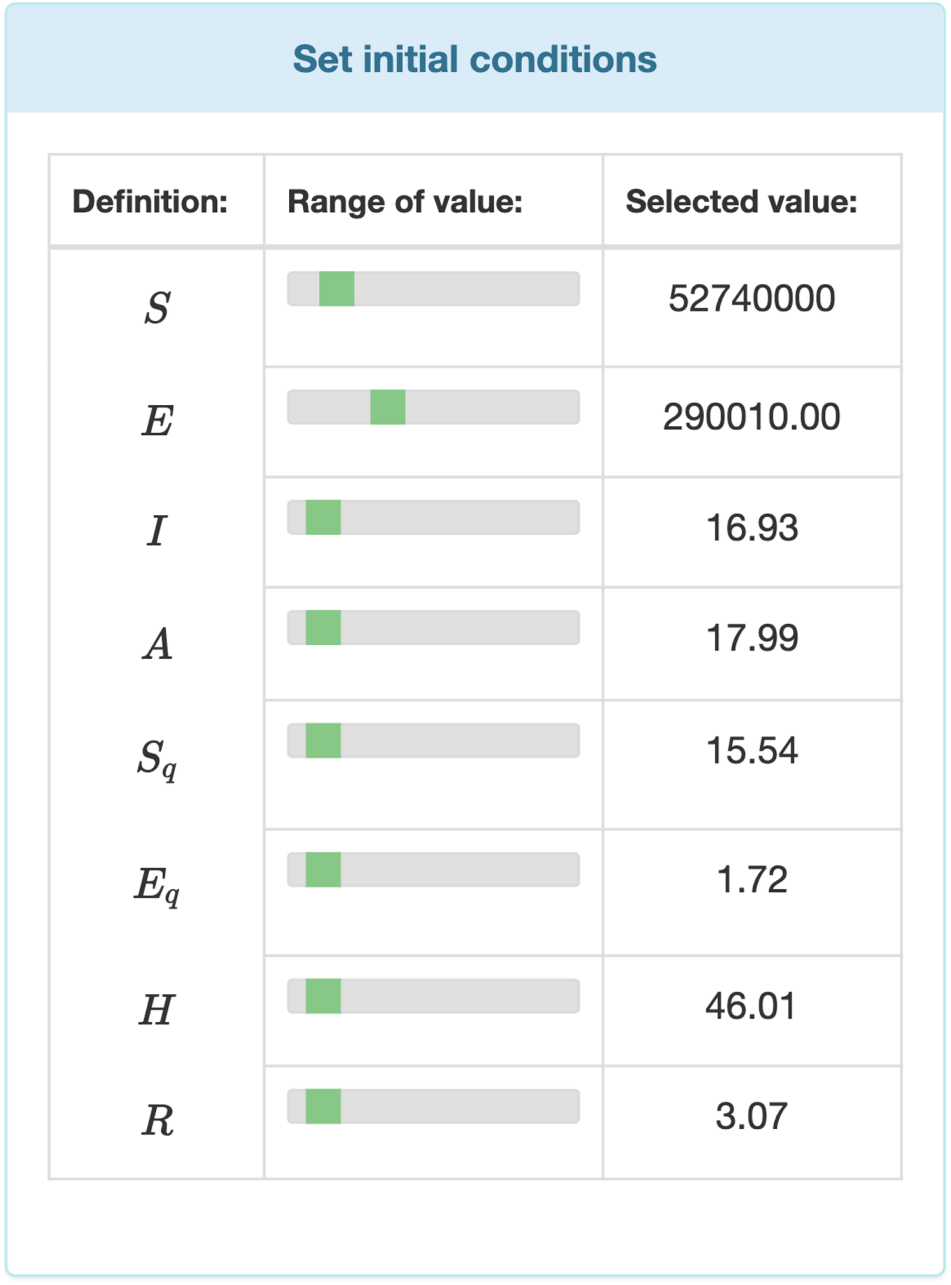
Input fields for the initial values of the differential equation system.

**Figure 4:**
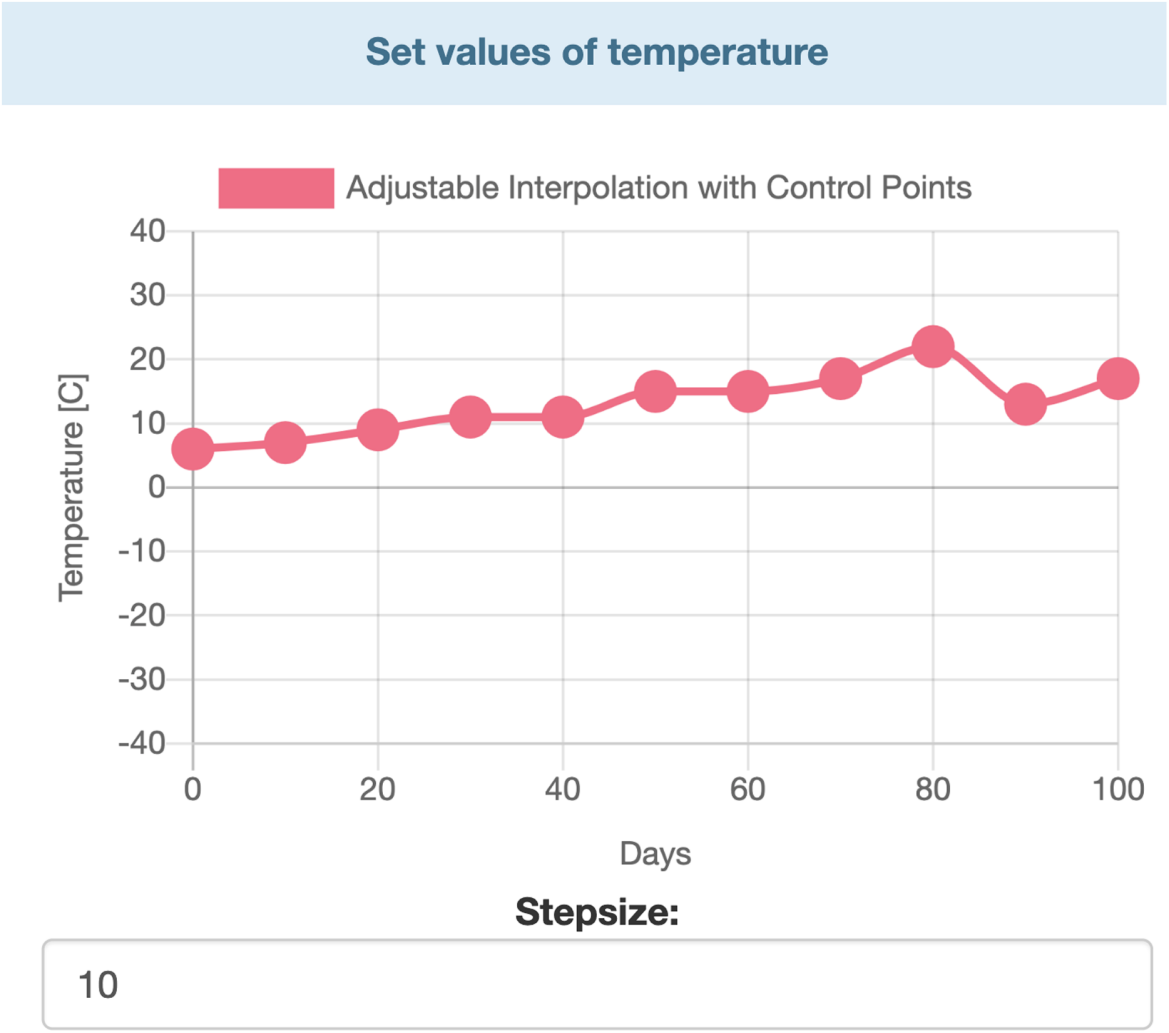
Interactive chart for temperature.

Data entry is further divided in two two major parts. Users can entry specific data by selecting values from ranges. Those parameters’ values which are not time dependent can be modified by range inputs (see Fig. 3). Parameters that vary over time, such as temperature, humidity, etc., are defined with the help of interactive charts, where users can place and move around so called “control points” (see Fig. 4). The points are linked using Cubic Spline Interpolation, which fits the given data by a piecewise polynomial functions avoiding overfitting Xie (1984). The resulting spline interpolation defines the parameters’ values over time.

Complementing the easy manual parameter definition, the visualization dashboard also provides a way to meticulously fine-tune the input data. The *Parameter import*/*export* section allows the user to import initial values and parameter data in the *JSON* format, and one can also export the current settings in the sam format. After an export, the parameters in the *JSON* can be modified at the desired precision, and then the user can proceed with the re-import of the *JSON* file.

**Figure 5:**
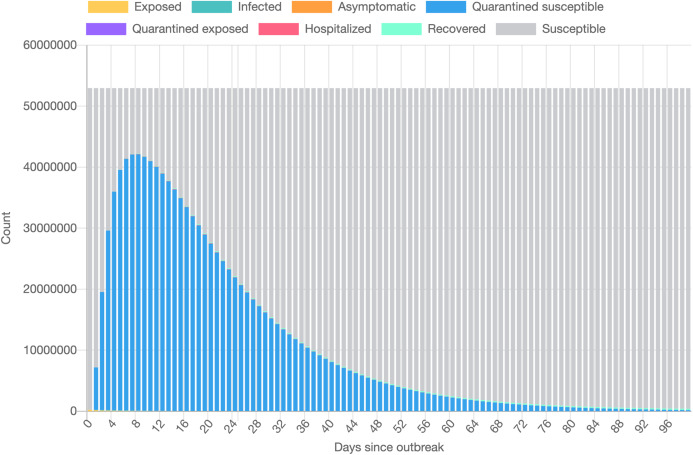
Data visualization based on the epidemiological model.

The data visualization is realized using the Chart.js open-source Javascript library (see Fig. 5). The user can choose which functions to visualize, making the diagram less crowded and more understandable.

The dashboard implements a “reactive” user interface, meaning that the user is immediately aware of the effect of each change, modification in the inputs. Therefore, in order to satisfy the real-time update constraints, it was paramount to be able to compute the model’s outputs very efficiently. The classic Runge–Kutta IV (RK4) method, implemented in the Runge–Kutta library (https://www.npmjs.com/package/runge-kutta), suited our needs.

## Results

The model parameter estimations are presented in Tables 3 and 4. Table 4 contains the approximated parameter values.

**Table 3:** Estimated initial values.

Functions	Estimated initial values (Northern Italy)	Estimated initial values (Southern Italy)
*E*	12.2097	1.5087
*I*	44.4572	1.3129
*A*	10.5571	1
*S*_*q*_	12.4293	10.3178
*E*_*q*_	1.5760	1
*H*	5.4811	1
*R*	0.0	0.0

**Table 4:** Parameter estimations.

Parameter	Estimated param. values (Northern Italy)	Estimated param. values (Southern Italy)
*c*_0_	16.5854	25.9026
*c*_*a*_	1.2799	1.25
*q*_1_	3.4073·10^−8^	1.1308·10^−7^
β	2.5623·10^−9^	4.0106·10^−9^
*q*_0_	2.1632·10^−9^	6.7791·10^−9^
σ	0.2102	0.2457
ϱ	0.98	0.98
θ	1.7539·10^−5^	2.6082·10^−5^
α*_b_*	0.0014	0.0031
ξ	0.0044	0.0023
*b*	0.99	0.99

**Figure 6:**
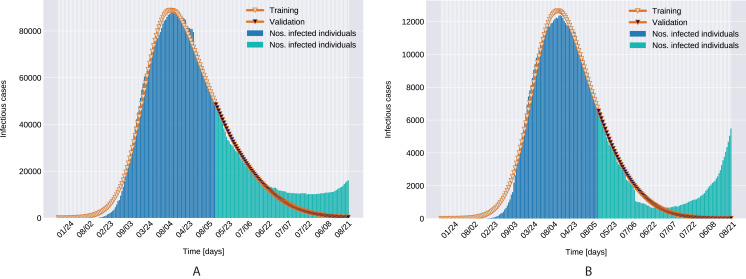
Infectious person count (blue & green bars) and the model’s data fitting (orange curves). (A) Northern Italy with 120 days training, 99 days validation. (B) Southern Italy with 120 days training and 99 days validation regions.

As depicted in Fig. 6, the estimated parameters provide a very good fit to the reported daily infectious cases data, both on the training and the beginning of the validation period.

**Figure 7:**
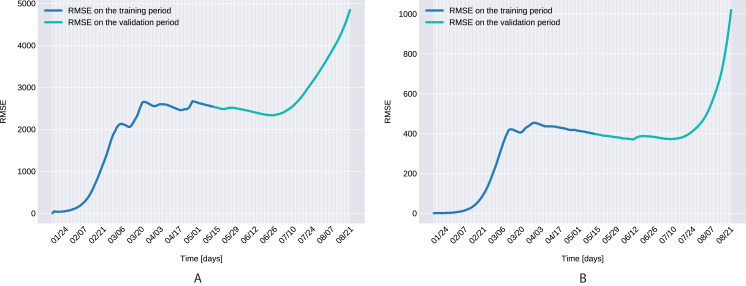
The Root Mean Square Error for the training (120 days) and validation (99 days) periods for for Northern Italy (A), and Southern Italy (B).

The Root Mean Square Error, defined as $RMS{E_d} = \sqrt {\textstyle{1 \over D}\sum\nolimits_{t = 1}^D {{(I(t) - \bar I(t))}^2}}$ with *D* denoting the number of days in the prediction range, ī(*t*) and *I*(*t*) being the actual and modeled active cases on the *t*-th day, is presented in Fig. 7 for both the training and validation period.

## Discussion

In this section we present some aspects of the model parameters.

**Figure 8:**
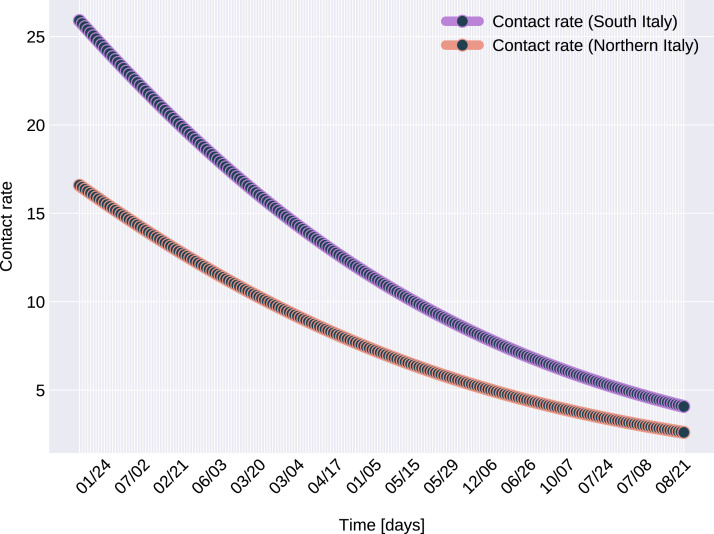
The graph of the function *c(t)*, for Northern and Southern Italy.

**Figure 9:**
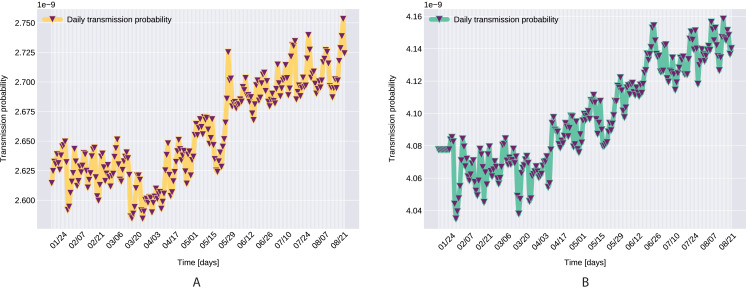
Transmission probability, Northern Italy (A), South Italy (B).

**Figure 10:**
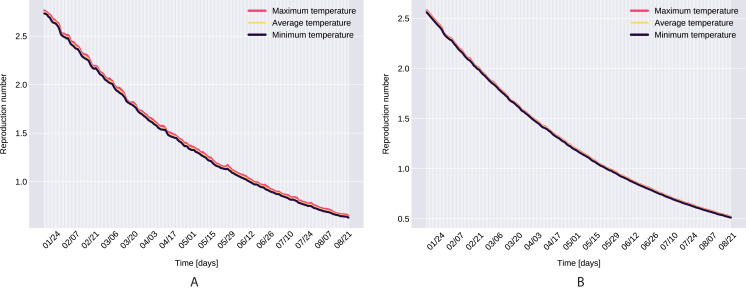
The graph of the function *R(t)*, Northern Italy (A), South Italy (B).

- Similarly to the results reported for Italy in Kozio, Stanisawski & Bialic (2020), the modeled transmission, depicted in Fig. 6, is higher than the actual one for the first 5 weeks, while in the second half of the validation period, the actual transmission is significantly higher than the modeled one. A possible explanation put forward in Kozio, Stanisawski & Bialic (2020) is that these discrepancies might arise from the absence or the relatively low daily tests in the beginning of the outbreak, respectively from the result of gradually easing the restrictions and the onset of the second wave.
- The Root Mean Square Error at the end of the training period is *RMSE*_120_ = 2,523 for Northern Italy, respectively *RMSE*_120_ = 396 for Southern Italy. The error is in the range of other studies. While the training was for 150 days and it comprised all regions of Italy, Kozio, Stanisawski & Bialic (2020) reported an *RMSE*_120_ of 3,283. As seen in Fig. 7, for both regions the *RMSE* stays at the same level, or even slightly decreases, in the first 60 days of the validation period. Towards the end of the validation period the *RMSE* quickly rises for both regions, as the model is not able to predict the onset of the second-wave.
- The proposed model assumes that the contact rate (Eq. (6)) is a monotonically decreasing function, therefore it can not account for multiple waves, that might occur after a relaxing of the social distancing measures. Several studies research the modeling possibilities of multi-wave epidemic outbreaks. The proposed models try to fit waves to the measured data, extrapolating potential outcomes and repeated outbreaks, nevertheless we can affirm, that they are not being able to predict future subepidemics, only fitting to past or ongoing waves and extrapolating them. A mechanistical model Camacho & Cazelles (2013) tries to benefit from the knowledge about immune response of infected individuals, specifically that there exists two types of immune responses, providing short term and long term immunity, thus opening a possibility for a reinfection. Another model evaluates regions based on a classification of the population for being susceptible in the first wave, or later waves. Findings of the research are, that geolocalization and traveling of individuals combined with delayed immune reactions can provide the basis of multiple waves Kaxiras & Neofotistos (2020). Our model can’t handle multi-wave pandemics, indeed. But the aim of the proposed model is rather to study an ongoing epidemic/subepidemic, providing a useful parameter array which helps identifying the possible directions of public reaction aiming the change of the given epidemic curve. Thus the following outbreaks, seasonal or induced by ineffective ending of the current outbreak (based on public health status, social migration, implemented public actions) are parametrized by the final status and outcomes of the previous wave. But, as the mentioned studies, we are not yet willing and ready to predict/extrapolate multiple waves ahead, with sufficient precision, based on the current epidemiological status. Though, for a future research direction we are considering a more complex model, which eventually forecasts or combines multi-wave situations.
- In the case of Northern Italy, the initial contact rate is slightly higher than in case of South Italy. It is possible that in that period tourism in Northern Italy was more prevalent than in South Italy due to winter activities. Looking at the approximated contact rate parameter and evolution of *c*(*t*) if Fig. 8 we can observe a quick drop. Italy officials have implemented strict social movement restrictions, which lead in time to a major decrease of the effective contact rate in the city. Home confinement led to even a 90% reduction of the contact rate. By elevating the contact rate to be a function, which decreases under the governmental actions’ influence, we obtain a shrinkage that reaches 50% in approximately 90 days and tends toward the minimum contact rate (*c*_*a*_), which is 92.29% smaller than the initial contact rate. Our model’s results seem to confirm the effectiveness of the measures implemented in Italy. As in both regions the contact rate converges to minimum contact rate (*c*_*a*_), we can confirm that preventative measures were successful.
  In other scenario, many asymptomatic or unknown infections are not considered by the official case counting, as they cannot be taken into evidence, see Liu et al. (2020d). Therefore, even when in reality the contact rate has dropped more, the percentage of unaccounted cases that go critical and need hospitalization, keep *I*(*t*) inflated. As the model has no awareness of unaccounted cases, it compensates the fast growth in *I*(*t*) by maintaining a high contact rate. Most probably, both factors play a role in the elevated *c*_*a*_ and *b* values.
- A pioneer step in epidemiological studies was the inclusion of two of the main environmental parameters in the process, temperature and humidity, as basic natural influencing factor for virus spreading (Lowen et al., 2007; He et al., 2013). Experiments conducted with the influenza virus on animals showed a connection between the virus transmission rate, temperature and humidity: decreasing temperature and increasing humidity leads to an increased virus transmission rate (Lowen et al., 2007). Lowen et al. (2007) included this theory in their *SIR* model using the temperature as a relaxing factor in the transmission probability time-dependent function. The median value for ξ in He et al. (2013) is 0.057. However, the present paper suggests that warmer temperatures slow COVID-19 transmission, but not significantly, as ξ values suggests both in case of Northern Italy (0.0044) and Southern Italy (0.0023). This affirmation is founded by studies such as Sehra et al. (2020), Gupta, Raghuwanshi & Chanda (2020). The introduction of time-dependent transmission probability β(*t*) increased our fit percentage to the empirical data and also proves that weather conditions do not have a significant effect to the spread of COVID-19 disease (the obtained graph of function β can be seen on Fig. 9).
  From the definition of the daily reproduction number (the obtained function can be seen on Fig. 10), we see that
  $$R(t)=\left[\frac{\rho\beta(t)c(t)(1-q(t))}{\delta_{I}+\gamma_{I}}+\frac{\theta(1-\rho)\beta(t)c(t)(1-q(t))}{\gamma_A}\right]S_0,$$
  We ran the approximation process for the given time period’s minimum, maximum, and average daily temperature and humidity respectively, which also suggests that temperature does not have a significant affect. However, as it takes its highest values when we use the minimal temperatures, bounds were set for daily reproduction number. Note that, our proposed parameter estimation method does not approximate the values of the parameter δ_*I*_, γ_*I*_. However, we can estimate the sum of δ_*I*_, γ_*I*_ (see Fig. 2), and for the value γ_*A*_ we used the value calculated in Tang et al. (2020a).
  As a final remark on *R*(*t*), the definition of the daily reproduction number for the model incorporating the disease induced mortality rate would be
  $$R_\alpha(t)=\left[\frac{\rho\beta(t)c(t)(1-q(t))}{\delta_{I}+\alpha+\gamma_{I}}+\frac{\theta(1-\rho)\beta(t)c(t)(2-q(t))}{\gamma_A}\right]S_0.$$
  Since α > 0, it is obvious that *R*_α_(*t*) ≤ *R*(*t*). The approximation process for the first wave (till 2 of July 2020) gives that
  $${\bar {\cal R}}_{N. {\rm Italy}} \leq 0.2369, \; {\bar {\cal R}}_{S. {\rm Italy}} \leq 0.1323.$$
- There are still many unknowns and uncertainty regarding SARS-CoV-2, including one of the key epidemiological parameters, the incubation period and its distribution. Correct assessment of the incubation period helps determine the appropriate duration of quarantine, and indicates how far contact tracing efforts of suspected individuals should go.
  In absence of data on the SARS-CoV-2 incubation period, initial parameter estimations for COVID-19 epidemiological models Tang et al. (2020a, 2020b, 2020c) have assumed incubation periods of SARS and MERS coronaviruses. Following upon Chowell et al. (2004), which determined a mean incubation period of 6.37 days for the 2002–2003 SARS outbreak, these studies used 7 days as the mean value.
  Field reports and later studies suggested shorter incubation periods, of 5.8 days (Backer, Klinkenberg & Wallinga, 2020) 5.2 days (Li et al., 2020b), 5 days (Zhou et al., 2020), 4 days (Read et al., 2020) and even 3 days (Guan et al., 2020; Lin et al., 2020). Currently, the WHO (https://www.who.int/news-room/q-a-detail/q-a-coronaviruses) states that “most estimates of th2e incubation period for SARS-CoV-2 range from 1–14 days, most commonly around 5 days.” The estimated parameter for the transition rate of exposed individuals to the infected class for the proposed model is 0.1706, resulting in a 5.86 days mean incubation period. This value almost matches the estimated value of 5.8 (Backer, Klinkenberg & Wallinga, 2020) gathered from the travelers with confirmed 2019-nCoV infection in the Wuhan, China region in the early stage of the outbreak.

**Figure 11:**
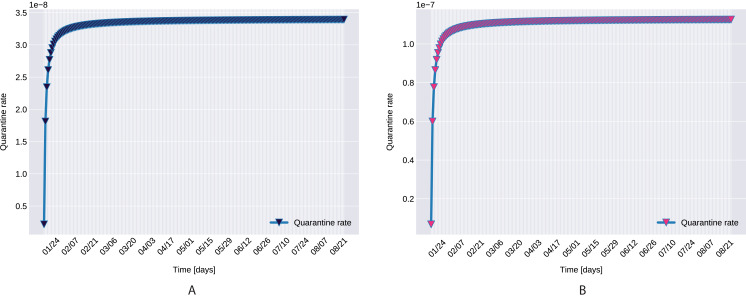
The graph of the function *q(t)*, Northern (A) and Southern Italy (B).

The proposed parameter estimation procedure measures error on *I*(*t*), therefore it can approximate correctly only the parameters that directly influence it, namely the ones related to contact rate, transmission probability, incubation period, probability of having symptoms among infected individuals. With more detailed data, with a breakdown of number of isolated individuals, number of deceased individuals per compartments, recovered individuals (not only from the hospitalized people) the error function could be extended to minimize errors also on these values, provide an even better insight into the epidemiological dynamics described by our proposed model.

## Conclusions

According to the WHO reports (www.who.int), time dependent mathematical models can help understand and predict the transmission of the outbreak, support the impact evaluation of the existing and future public health interventions, and provide a better grasp on the severity of ongoing outbreaks. Factors that have major influence the spread of SARS-CoV-2 may evolve and change over time. To account for such changes, in this paper we propose a time dynamic, temperature dependent SEIR-type extended epidemiological model.

The paper also proposes an Iterated Particle Swarm Optimization (IPSO) method, that efficiently explores only the vicinity of already established local optima. The method searches iteratively around better and better local optima and it is able to accurately estimate all the required parameters of the model in a reasonable running-time.

By matching the historical data closely, the model can provide more accurate near-projection of the trend and peak time estimations. Analyzing and comparing the estimated contact rate and the estimated transmission probability from different outbreaks might also advance the understanding of the unfolding trends.

## Additional Information and Declarations

### Data Availability

The following information was supplied regarding data availability: Initial condition for the proposed model is available at GitHub: https://github.com/pcm-dpc/COVID-19. The dataset provided byJohns Hopkins University Center is also available at GitHub: https://github.com/CSSEGISandData/COVID-19. PSO is available at Distributed Evolutionary Algorithms in Python (DEAP): https://deap.readthedocs.io/en/master/examples/pso_basic.html.
